# The Biochemical Role of the Human NEIL1 and NEIL3 DNA Glycosylases on Model DNA Replication Forks

**DOI:** 10.3390/genes10040315

**Published:** 2019-04-23

**Authors:** Mustafa S. Albelazi, Peter R. Martin, Soran Mohammed, Luciano Mutti, Jason L. Parsons, Rhoderick H. Elder

**Affiliations:** 1 Biomedical Research Centre, School of Environment and Life Sciences, Peel Building, University of Salford, Salford M5 4NT, UK; m.s.albelazi@edu.salford.ac.uk (M.S.A.); peter.martin@icr.ac.uk (P.R.M.); soran.mohammed@soton.ac.uk (S.M.); luciano.mutti@hotmail.it (L.M.); 2 Chemical Biology, Diagnostics and Therapeutics Group, Chemistry Faculty, University of Southampton, Southampton SO17 1BJ, UK; 3 Sbarro Institute for Cancer Research and Molecular Medicine, Center for Biotechnology, College of Science and Technology, Temple University, Philadelphia, PA 19122, USA; 4 Cancer Research Centre, Department of Molecular and Clinical Cancer Medicine, University of Liverpool, 200 London Road, Liverpool L3 9TA, UK; j.parsons@liverpool.ac.uk

**Keywords:** base excision repair, DNA damage, DNA repair, DNA replication, NEI-like DNA glycosylases

## Abstract

Endonuclease VIII-like (NEIL) 1 and 3 proteins eliminate oxidative DNA base damage and psoralen DNA interstrand crosslinks through initiation of base excision repair. Current evidence points to a DNA replication associated repair function of NEIL1 and NEIL3, correlating with induced expression of the proteins in S/G2 phases of the cell cycle. However previous attempts to express and purify recombinant human NEIL3 in an active form have been challenging. In this study, both human NEIL1 and NEIL3 have been expressed and purified from *E. coli*, and the DNA glycosylase activity of these two proteins confirmed using single- and double-stranded DNA oligonucleotide substrates containing the oxidative bases, 5-hydroxyuracil, 8-oxoguanine and thymine glycol. To determine the biochemical role that NEIL1 and NEIL3 play during DNA replication, model replication fork substrates were designed containing the oxidized bases at one of three specific sites relative to the fork. Results indicate that whilst specificity for 5- hydroxyuracil and thymine glycol was observed, NEIL1 acts preferentially on double-stranded DNA, including the damage upstream to the replication fork, whereas NEIL3 preferentially excises oxidized bases from single stranded DNA and within open fork structures. Thus, NEIL1 and NEIL3 act in concert to remove oxidized bases from the replication fork.

## 1. Introduction

It is estimated that in each cell approximately 10,000 DNA bases are chemically modified every day [1]. Cells have evolved complex DNA repair mechanisms to respond to specific DNA damage, to prevent mutagenesis and carcinogenesis [2,3,4]. Base excision repair (BER) is a highly conserved DNA repair mechanism responsible for the removal of chemically altered bases, and the repair of abasic sites and single-strand breaks [5,6]. In human cells, removal of damaged bases proceeds via one of eleven lesion-specific DNA glycosylases that cleave the N-glycosylic bond between the deoxyribose sugar and modified base [7]. DNA glycosylases are crucial for maintaining genome stability and multiple lines of evidence identify roles within disease development and maintenance, including neurodegeneration, autoimmunity and cancer [8,9,10]. Therefore, a fundamental understanding of the biochemical mechanisms of DNA glycosylases in base damage removal within specific DNA substrates generated during normal physiology is essential in elucidating the cellular roles that they play in vivo.

Monofunctional DNA glycosylases, such as uracil DNA glycosylase, initiate BER through the hydrolysis of the N-glycosylic bond generating an apurinic/apyrimidinic (AP) site. The AP-site is then processed by AP-endonuclease-1 (APE1) that cleaves the phosphodiester backbone producing a 5′-deoxyribose phosphate (dRP) and a 3′-hydroxyl group [11,12]. In short-patch BER, DNA polymerase β (Pol β) then removes the sugar phosphate through its deoxyribose phosphate lyase activity, followed by single base gap filling [13,14]. The remaining nick in the phosphodiester backbone is then sealed by DNA ligase III under the coordination of the scaffold protein XRCC1 [15,16]. This is termed short-patch BER through which the majority of DNA base lesions are processed [17]. An alternative method, long-patch BER, employs displacement DNA synthesis using DNA replicative enzymes to replace the damaged nucleotide, and has recently been implicated in the repair of telomeric DNA sequences in association with NEIL3 [18]. Bifunctional DNA glycosylases that possess an associated AP lyase activity have two mechanisms of action. In the first, the phosphodiester backbone is incised 3′ to the AP site producing a 5′-phosphate and a 3′-polyunsaturated aldehyde moiety (β-elimination), that is further processed by APE1 generating a 3′-hydroxyl group. Alternatively, DNA glycosylases that possess β,δ-lyase activity cleave the phosphodiester backbone in two places producing 5′-phosphate and 3′-phosphate ends. The 3′-phosphate group is then processed by polynucleotide phosphatase/kinase to produce the necessary 3′-hydroxyl end for gap filling by Pol β [19].

**Figure 1:**
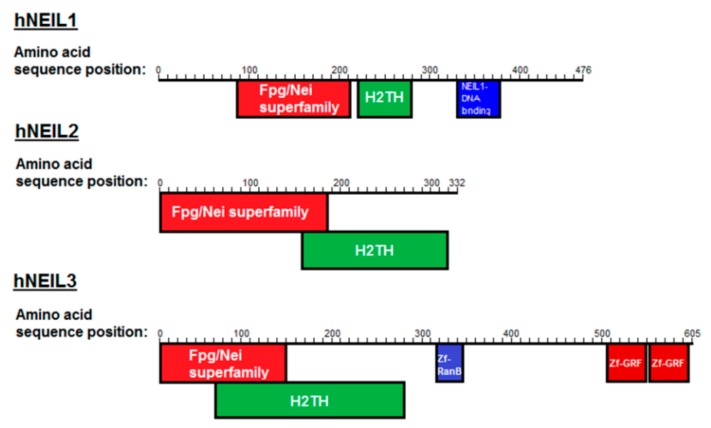
Conserved domains of the Nei-like DNA glycosylases (hNEIL1, hNEIL2 and hNEIL3). Shown are the Fpg/Nei superfamily domains, helix-two-turn helix (H2TH) domains, zinc finger RanB domains (Zf-RanB) domains and zinc finger-GRF domains (Zf-GRF).

The endonuclease VIII family of DNA glycosylases are of particular interest, as mammalian cells have evolved three distinct Nei-like DNA glycosylases known as NEIL1, NEIL2 and NEIL3 (Figure 1; [20]). This family of DNA glycosylases share an N-terminal DNA glycosylase domain and a helix-two-turn helix (H2TH) DNA binding domain. NEIL3 is unique in that it possesses an extended C-terminal domain, containing three additional zinc finger binding domains. Despite this, NEIL1 and NEIL3 have a broad substrate preference showing significant overlap and complementation in their lesion processing [21,22]. However human NEIL1 (hNEIL1) has a general preference for oxidized bases (with the notable exception of 8-oxoguanine (8-oxoG)) in double-stranded (ds) DNA exhibiting a β,δ-lyase activity, while human NEIL3 (hNEIL3) has been shown to have a preference for single-stranded (ss) DNA, incising the DNA through a weak β-lyase activity [23]. Both hNEIL1 and hNEIL3 though have high levels of activity on the further oxidation products of 8-oxoG, spiroiminodyhidantoin (Sp) and guanidinohydantoin (Gh) [20,21,22,23,24]. hNEIL1 and the mouse ortholog of NEIL3 have also been demonstrated to excise Sp and Gh lesions in G-quadruplex structures with a preference for DNA in telomeric context, suggestive of structural preferences of the NEIL glycosylases [18,25,26]. Interestingly, hNEIL1 and hNEIL3 have been reported to be cell-cycle regulated, with expression peaking at S/G2, while hNEIL2 appears to be constitutively expressed [27,28].

hNEIL1 has been associated with pre-replicative repair and is suggested to stall the replisome on detection of 5-hydroxyuracil (5-OHU) in ssDNA, promoting fork regression and leading to the reannealing of the DNA and eventual removal of the lesion by the DNA glycosylase (Hegde et al., 2013; Rangaswamy et al., 2017). hNEIL1 has also been observed to co-localize with DNA replication proteins at sequences within the replicating genome [29,30]. hNEIL3 was demonstrated to co-precipitate with the replisome, while mitotic defects in the form of telomere bridges have been observed in NEIL3^-/-^ MEFs, hNEIL3 knockdown HCT116 colorectal cancer cells and DNA glycosylase null primary human D132V mutant fibroblasts [18,31]. More recently, hNEIL1 and hNEIL3 were found to process psoralen mono-adducts and interstrand DNA crosslinks (ICLs) in three and four-stranded DNA structures and unhooking ICLs at replication fork structures [24,32,33]. Therefore, cumulatively, this evidence advocates a role for hNEIL1 and hNEIL3 in DNA replication associated DNA repair and coordination, in actively dividing cells. However, this requires further confirmation and particularly the substrate specificity for hNEIL1 and hNEIL3 on oxidized DNA bases within model fork substrates needs more detailed experimental analysis.

While hNEIL1 and hNEIL2 have been purified from *E. coli* and subsequently characterized using oligonucleotide substrates, hNEIL3 has been a particular challenge due to its inherent instability [21,22]. However, the Wallace group made significant advances in the production of an active NEIL3 protein, designing a modified pET expression system to successfully produce catalytically active forms of the N-terminal DNA glycosylase domain of the mouse and human NEIL3 proteins [21]. It was determined that secondary structure formation of mRNA and the lack of post-translational methionine processing by *E. coli*, detrimentally affected active recombinant NEIL3 protein expression. Thus, a bicistronic vector was designed that co-expresses a modified *E. coli* methionine aminopeptidase (EcoMapY168A) fused to the N-terminus of NEIL3. In this study, this vector was used to express full-length hNEIL3 (hNEIL3^FL^) and used in activity assays in comparison with recombinant hNEIL1 to probe the distinct biochemical activities of the enzymes within the context of a model DNA replication fork.

## 2. Materials and Methods

### 2.1. Materials

The pET30a-hNEIL1 and pETDuet2-*EcoMap*-ORF6-MmNEIL3 bacterial expression plasmids were kind gifts from Susan Wallace and are as previously described [21,34]. The bicistronic expression vector pETDuet2 was used to generate the hNEIL3 expression vector pETDuet2-*EcoMap*-ORF6-hNEIL3^FL^. Briefly, due to an internal NdeI restriction site within the hNEIL3 cDNA (at position 864), the pETDuet2-*EcoMap*-ORF6-MmNEIL3 vector was first digested with NdeI (New England Biolabs, Hitchin, UK) and then incubated with mung bean nuclease to yield a blunt end. The ORF6-MmNEIL3 sequence was then removed by digestion with XhoI (New England Biolabs, Hitchin, UK) and the linearized pETDuet2 vector gel purified. ORF6-hNEIL3^FL^ DNA sequences with an additional thymidine nucleotide at the 5′-end and a XhoI restriction site at the 3′ end was obtained by PCR, digested with XhoI and ligated into the prepared pETDuet2 sequence. This facilitated the generation of the pETDuet2-*EcoMap*-ORF6-hNEIL3 expression vector containing hNEIL3^FL^ cDNA sequences. *E coli* endonuclease III (Nth) and endonuclease VIII (Nei) were from New England Biolabs (Hitchin, UK).

### 2.2. DNA substrates

The 39-mer oligonucleotide (5′-ATCTACCGAGTCCGTCCGAXCACGCTTATTGGCTACCGA-3′; where X is equivalent to either 5-OHU, 8-oxoG or thymine glycol; Tg) and containing either a 5′-Alexa Fluor 680 or IR Dye 800 fluorescent label was used as the ssDNA substrate. The complementary strand (5′-TCGGTAGCCAATAAGCGTGYTCGGACGGACTCGGTAGAT-3′; where Y is equivalent to guanine, cytosine or adenine opposite 5-OHU, 8-oxoG or Tg, respectively) was used to create the dsDNA substrate. For creation of the replication fork substrates, the following oligonucleotides were used (5’-GAATGCATTCCGCCATCGAGTCGGACGGACTCGGTAGAT-3′ for the fork; 5′-GAATGCATTCCGCCACGTGGTCGGACGGACTCGGTAGAT-3′ for fork+4; 5′-GAATGCATTCCGCCATCGATATCGACGGACTCGGTAGAT-3′ for fork-4). All oligonucleotides were HPLC purified and synthesized by IDT Technologies (Leuven, Belgium).

### 2.3. Expression and Purification of hNEIL1 and hNEIL3

Recombinant C-terminal His-tagged hNEIL1 was purified as previously described [35]. His-tagged hNEIL3^FL^ was similarly transformed and overexpressed in Rosetta 2 (DE3)pLysS *E. coli* (Merck-Millipore, Watford, UK) in Luria-Bertani (LB) media containing 50 µg/mL ampicillin, 33 µg/mL chloramphenicol and 0.1% glucose, although protein expression was induced by the addition of 1 mM isopropyl β-D-1-thiogalactopyranoside (IPTG) at 16 °C for 20 h in a shaking incubator. All subsequent steps were conducted at 4 °C. Cells were centrifuged (8000 rpm for 10 min), pellets resuspended in lysis buffer (25 mM Tris-HCl pH 8.0, 500 mM NaCl, 5% glycerol) containing 1 µg/mL protease inhibitors (aprotinin, pepstatin, leupeptin and chemostatin) and 1 mM phenylmethylsulfonyl fluoride (PMSF), sonicated and centrifuged (25,000 rpm for 20 min). The supernatant was filtered using 1 µm and then 0.45 µm syringe filters (Merck-Millipore, Watford, UK) and hNEIL1 and hNEIL3^FL^ were both purified using HisTrap chromatography (GE Healthcare, Little Chalfont, UK) using an imidazole gradient (5–500 mM) in lysis buffer. Fractions containing the proteins were identified by 10% SDS-PAGE and Instant Blue staining (Expedeon Ltd, Cambridge, UK) and buffer exchanged into storage buffer (50 mM Tris-HCl pH 8.0, 50 mM NaCl, 1 mM EDTA, 10% glycerol) using Amicon Ultra-15 centrifugal filter units (Merck-Millipore, Watford, UK). hNEIL3^FL^ underwent a second purification step using a Mono S chromatography (GE Healthcare, Little Chalfont, UK) and salt gradient elution (50–1000 mM KCl). Purified hNEIL1 and hNEIL3^FL^ were aliquoted and stored at −80 °C prior to use.

### 2.4. DNA Glycosylase Activity Assays

To analyze DNA glycosylase cleavage activity, hNEIL1 and hNEIL3^FL^, diluted in 25 mM HEPES pH 7.9, 100 mM KCl, 12 mM MgCl_2_, 1 mM EDTA, 17% glycerol and 2 mM DTT, were incubated with 5 nM of oligonucleotide substrate molecules for 30 min at 30 °C in reaction buffer (40 mM HEPES pH 7.8, 5 mM MgCl_2_, 0.1 mM EDTA, 0.5 mM DTT, 2 mM ATP, 0.1 mg/mL BSA) in a final volume of 10 µL. This is a standard reaction buffer used in our laboratory for BER reactions, although ATP and MgCl_2_ are not required for DNA glycosylase activity. Reactions were stopped by the addition of an equal volume of formamide loading dye (95% formamide, 0.05% bromophenol blue) and heated to 95 °C for 5 min. Reaction products were separated by 10% denaturing-PAGE and subsequently quantified using the Odyssey Image Analysis System (Li-Cor Biosciences, Cambridge, UK). For sodium borohydride trapping of hNEIL3, reactions were supplemented with 100 mM sodium borohydride and the reaction performed at 30 °C for 30 min. The reaction was stopped by the addition of 10 µL of 3× SDS-PAGE sample buffer (25 mM Tris-HCl pH6.8, 2.5% 2-mercaptoethanol, 1% SDS, 10% glycerol, 1 mM EDTA and 0.05% bromophenol blue) and heated to 95 °C for 5 min. Samples were separated by 10% Tris-Glycine SDS-PAGE and the gel was visualized using the Odyssey Imaging Analysis System.

## 3. Results

### 3.1. Purification of hNEIL3^FL^

**Figure 2:**
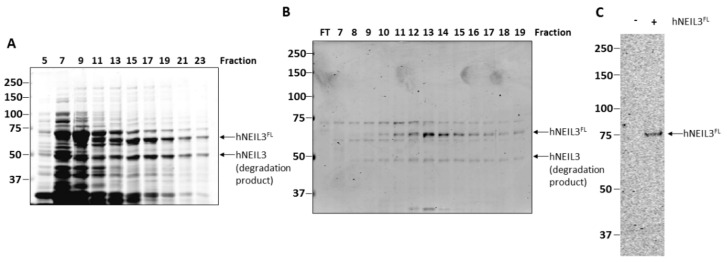
Purification of hNEIL3^FL^ as an active DNA glycosylase/lyase. Analysis of protein fractions generated by sequential (**A**) HisTrap chromatography and (**B**) Mono S chromatography of lysates from *E coli* overexpressing hNEIL3^FL^ by SDS-PAGE and Instant Blue protein staining. (**C**) Sodium borohydride trapping assay of hNEIL3^FL^ with a ssDNA substrate containing 5-OHU and analysis by SDS-PAGE.

hNEIL3^FL^ was expressed in Rosetta 2(DE3)pLysS *E. coli* cells and purified by HisTrap chromatography, although protein expression was found to be relatively weak and the protein appeared susceptible to degradation (Figure 2A), as confirmed by western blotting using anti-his tag antibodies (Figure S1). Therefore, hNEIL3^FL^ was further purified using ion exchange (Mono S) chromatography, after which the protein was found to be of sufficient purity (>80%) for use in biochemical activity assays (Figure 2B). A band of around 75 kDa was present in all fractions following MonoS chromatography, however, this polypeptide did not display the same elution profile as hNEIL3^FL^ and fractions were pooled to minimize this contamination. To ensure that the hNEIL3^FL^ protein was active and to exclude any potential contaminating bacterial DNA glycosylase/lyases (Fpg, Nth and/or Nei), a sodium borohydride trapping assay was undertaken using a ssDNA substrate oligonucleotide containing 5-OHU. This covalently links the DNA glycosylase with β-lyase activity to the incised substrate, allowing the DNA-protein complex to be analyzed by SDS-PAGE. Utilizing this assay, we observed only a single band when hNEIL3^FL^ protein was present in the reaction mix, and that the band obtained was of the expected size for hNEIL3 complexed to DNA (Figure 2C). No additional contaminating bands equivalent to Nei and Fpg proteins of *E. coli* (30.2 kDa, 31.4 kDa respectively) were observable, and nevertheless these would not be expected to be in large excess in comparison to the hNEIL3^FL^ overexpressed protein following chromatography purification. This experiment, however, also confirmed that the hNEIL3^FL^ protein was functionally active as a DNA glycosylase/lyase.

### 3.2. Assessment of Protein Stability of hNEIL3^FL^

**Figure 3:**
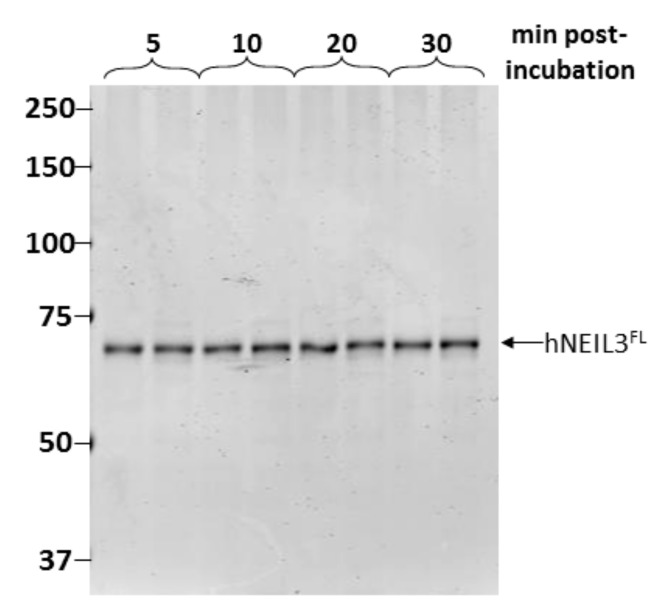
hNEIL3^FL^ is stable under reaction conditions. Duplicate samples of NEIL3^FL^ were incubated at 30 °C for 5–30 min in reaction buffer, and samples analyzed by SDS-PAGE and Instant Blue protein staining.

As NEIL3 has been reported to be unstable, we then determined the physical stability of the protein under our standard assay conditions. Thus, aliquots of MonoS purified hNEIL3^FL^ were prepared for assay analysis by buffer exchange and concentration and incubated for up to 30 min at 30 °C. This step appeared to have removed the contaminating proteins or that these were unstable following incubation. But more importantly this revealed that hNEIL3^FL^ was remarkably stable under these conditions, with only a single major band corresponding to the expected size of hNEIL3^FL^ present in each duplicate reaction (Figure 3).

### 3.3. Activity of hNEIL1 and hNEIL3^FL^ on ssDNA and dsDNA Substrates

**Figure 4:**
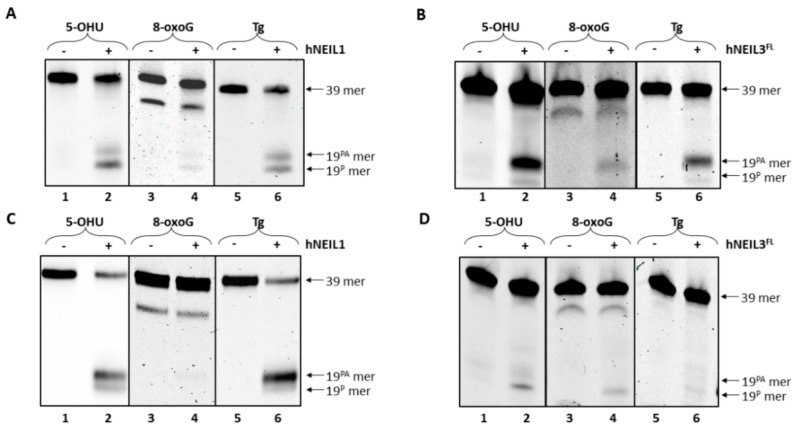
hNEIL1 and hNEIL3^FL^ demonstrate preferential cleavage of 5-OHU and Tg from dsDNA and ssDNA, respectively. (**A**) hNEIL1 and (**B**) hNEIL3^FL^ were incubated with ssDNA containing either 5-OHU, 8-oxoG or Tg and products analyzed by denaturing PAGE. (**C**) hNEIL1 and (**D**) hNEIL3^FL^ were incubated with dsDNA containing either 5-OHU, 8-oxoG or Tg and products analyzed by denaturing PAGE.

We next analyzed the biochemical specificity of both hNEIL1 and hNEIL3^FL^ enzymes for three different oxidized bases (5-OHU, 8-oxoG and Tg) in either ssDNA or dsDNA. Both hNEIL1 and hNEIL3^FL^ were found to incise ssDNA substrates containing a single 5-OHU or Tg residue, although hNEIL1 principally used β,δ-elimination (Figure 4A, lanes 2 and 6) whereas hNEIL3^FL^ utilized β-elimination (Figure 4B, lane 2 and 6). hNEIL1 was observed to display minimal activity against 8-oxoG containing ssDNA (Figure 4A, lane 4) whereas hNEIL3^FL^ showed weak β-lyase activity against this substrate (Figure 4B, lane 4). In contrast with dsDNA, hNEIL1 showed increased incision activity on substrates containing 5-OHU and Tg, (Figure 4C, lane 2 and 6), whereas hNEIL3^FL^ exhibited only minimal activity on 5-OHU and very little activity against Tg (Figure 4D, lanes 2 and 6). Similar to the results using ssDNA, hNEIL3^FL^ showed weak incision activity for 8-oxoG within dsDNA (Figure 4D, lane 4), whereas hNEIL1 displayed very minimal activity on this substrate (Figure 4C, lane 4).

### 3.4. Activity of hNEIL1 and hNEIL3^FL^ on Model DNA Replication Fork Substrates

Current evidence suggests that hNEIL1 and hNEIL3 initiate repair during DNA replication, therefore we generated a set of three model DNA replication fork structures to analyze comparative biochemical activity of the two DNA glycosylases. These substrates contained the oxidative lesions 5-OHU, 8-OxoG or Tg at one of three positions, either four nucleotides from the replication fork junction (−4) and within ssDNA, at the site of the fork junction (fork) and four nucleotides upstream from the replication fork (+4) within dsDNA (Table 1).

#### 3.4.1. Activity of hNEIL1 on Model DNA Replication Fork Substrates

**Figure 5:**
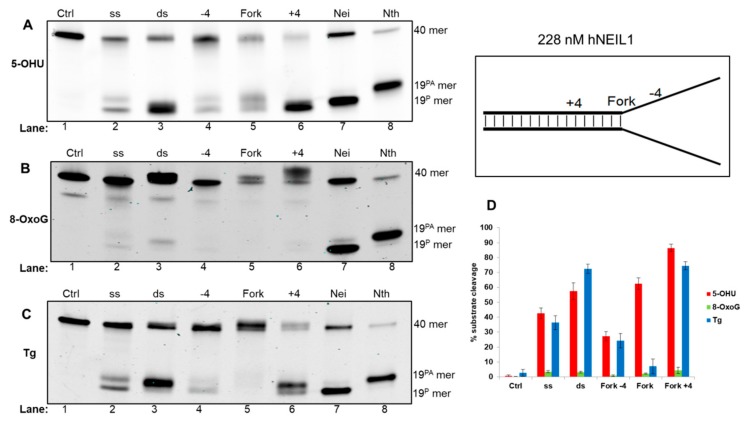
hNEIL1 shows a preference for cleavage of 5-OHU and Tg within dsDNA relative to the replication fork. hNEIL1 was incubated with oligonucleotide substrates containing either (**A**) 5-OHU, (**B**) 8-oxoG or (**C**) Tg and products analyzed by denaturing PAGE. (**D**) Quantification of substrate cleavage by hNEIL1. Bars show the mean ± standard error of four-five separate experiments. Control (Ctrl) refers to reaction in the absence of protein and the bacterial Nei and Nth enzymes were incubated with ssDNA containing substrates to show products of β,δ-elimination (19^P^) and β-elimination (19^PA^), respectively.

Analysis of reaction products in the presence of hNEIL1 with 5-OHU containing oligonucleotide substrates revealed a preference for dsDNA, with efficient cleavage of the dsDNA and fork+4 substrates via β,δ-elimination, whereas cleavage of 5-OHU in ssDNA and the fork−4 substrate was significantly reduced (Figure 5A, lanes 2–6; and Figure 5D). hNEIL1 also displayed reduced cleavage activity against the fork substrate when compared to the fork+4 and dsDNA, although a smeared DNA product, representative of the degradation of an apurinic site generated after monofunctional DNA glycosylase activity was observed (Figure 5A, lane 5). The bacterial DNA glycosylases Nei and Nth were used to demonstrate the relative migration of products generated via β,δ-elimination and β-elimination, respectively (Figure 5A, lanes 7 and 8). Analysis of 8-oxoG containing substrates incubated with hNEIL1 revealed minimal activity on all substrates analyzed, although very weak activity was observed on dsDNA (Figure 5B, lane 3). Incubation of hNEIL1 with Tg DNA substrates revealed efficient cleavage of the dsDNA substrate as well as the fork+4 substrate via β-elimination (Figure 5C, lanes 3 and 6). Comparatively, there was reduced efficiency of incision of the Tg within ssDNA by hNEIL1, and more so with the fork-4 substrate, whereas interestingly Tg in the fork substrate was not excised (Figure 5C, lanes 2, 4 and 5).

#### 3.4.2. Activity of hNEIL3^FL^ on Model DNA Replication Fork Substrates

**Figure 6:**
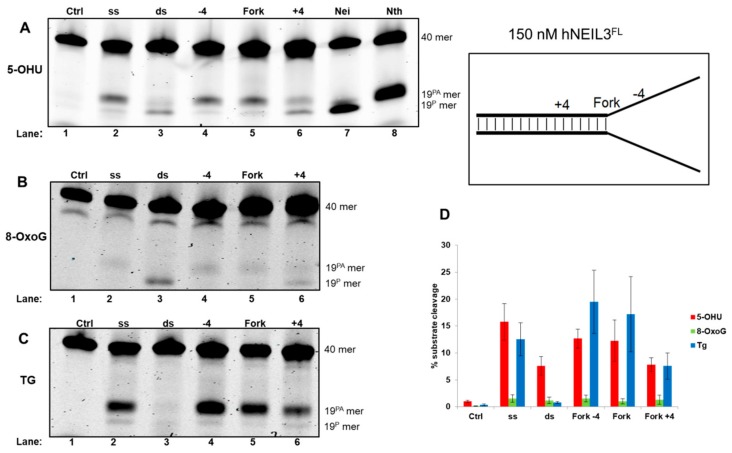
hNEIL3^FL^ shows a preference for cleavage of 5-OHU and Tg within ssDNA relative to the replication fork. hNEIL3^FL^ was incubated with oligonucleotide substrates containing either (**A**) 5-OHU, (**B**) 8-oxoG or (**C**) Tg and products analyzed by denaturing PAGE. (**D**) Quantification of substrate cleavage by hNEIL3^FL^. Bars show the mean ± standard error of four-five separate experiments. Control (ctrl) refers to reaction in the absence of protein and the bacterial Nei and Nth enzymes were incubated with ssDNA containing substrates to show products of β,δ-elimination (19^P^) and β-elimination (19^PA^), respectively.

Analysis of reaction products following hNEIL3^FL^ incubation with 5-OHU containing oligonucleotide substrates, underlined the overlapping substrate preference with hNEIL1. Thus, hNEIL3^FL^ displayed increased activity, largely on ssDNA substrates, and cleaved the fork−4 and fork substrate with greater efficiency than the fork+4 substrate (Figure 6A, lanes 2, and 4–6). hNEIL3^FL^ mainly displayed β-elimination activity against these substrates, in comparison to hNEIL1 that largely performed β,δ-elimination. However interestingly, hNEIL3^FL^ excises the 5-OHU lesion in dsDNA and the +4 fork structure, albeit with reduced efficiency, utilizing β,δ-elimination activity (Figure 6A, lane 3 and 6). Incubation of hNEIL3^FL^ with 8-oxoG containing DNA substrates displayed minimal levels of activity, although weak β,δ-elimination activity was observed with the dsDNA substrate (Figure 6B, lane 3). Analysis of hNEIL3^FL^ cleavage activity against Tg containing DNA oligonucleotide substrates revealed a preference for Tg in a ssDNA context. Indeed, robust activity on ssDNA and fork-4 substrates was observed (Figure 6C, lanes 2 and 4). hNEIL3^FL^ was also able to process the fork substrate relatively proficiently, however in comparison, incision of the fork+4 substrate was significantly reduced (Figure 6C, lanes 5 and 6). Interestingly with the Tg substrates, hNEIL3^FL^ displayed consistent β-elimination activity, unlike that observed for 5-OHU.

## 4. Discussion

In this study, recombinant human NEIL1 and NEIL3 DNA glycosylases were expressed and purified from *E. coli* cells and subsequently characterized using ssDNA, dsDNA and model DNA replication fork substrates containing one of three oxidized bases placed at site specific positions. Given previous evidence that the expression of hNEIL1 and hNEIL3 is overlapping during S-phase of the cell cycle, this suggested a role for these DNA glycosylases in initiating BER during DNA replication. We therefore speculated that hNEIL1 and hNEIL3 might have overlapping but distinct biochemical activities on DNA substrates mimicking those generated during DNA replication, specifically in relation to a replication fork.

Here we show that hNEIL1 preferentially cleaves 5-OHU and Tg DNA lesions in dsDNA and in dsDNA close to the replication fork, while displaying reduced levels of activity at the fork junction and more so in ssDNA. In contrast, hNEIL3^FL^ cleaves at 5-OHU and Tg preferentially within ssDNA and in ssDNA in one arm of the replication fork. This result is especially striking for Tg, where virtually no incision activity was observed for the lesion in the dsDNA substrate. Both hNEIL1 and hNEIL3 DNA glycosylases present extremely weak cleavage activity on the relatively abundant cellular oxidized base 8-oxoG, despite high levels of DNA glycosylase/lyase activity on the further oxidation products of 8-oxoG, Sp and Gh [22,24]. Whilst hNEIL3^Fl^ appeared to display comparatively reduced DNA glycosylase activity in comparison to hNEIL1, this is likely due to issues associated with expression of fully active hNEIL3^FL^ in *E. coli*, due to incorrect folding of the protein and inefficient removal of the N-terminal methionine residue, even in the presence of the modified aminopeptidase [21]. As previously described, the inherent instability of hNEIL3^FL^ may also have affected activity levels throughout this study, although no gross degradation of the protein was observed in our study and in others [22,23]. Nevertheless, our data are supportive of roles for both hNEIL1 and hNEIL3 in processing of oxidized DNA bases generated during DNA replication, particularly in a dsDNA and ssDNA context, respectively.

hNEIL1 has been suggested to possess a “cowcatcher” role in the presence of the replisome, with a mainly pre-replicative association, promoting fork regression and reformation of dsDNA required for cleavage by the enzyme [29,30]. We observed that hNEIL1 cannot efficiently process 5-OHU and Tg at the fork junction, however an increase in cleavage efficiency was observed within dsDNA close to the replication fork junction that could mimic a structure representing a regressed DNA replication fork. Therefore, while our data are supportive of this “cowcatcher” role, further probing of the positional preferences of hNEIL1 is required in the presence of the replisome to provide insight into the requirement for hNEIL1 activity close to the replication fork.

A role for hNEIL3 at the DNA replication fork was also recently described, indicating that hNEIL3 is fundamental in the protection of newly synthesized DNA and lack thereof resulted in an increase in DNA double-strand breaks, possibly as result of replication fork collapse [36]. Biochemical data presented here indicates that hNEIL3 is the primary Nei-like DNA glycosylase associated with the excision of oxidized bases within ssDNA and dsDNA at the replication fork, while NEIL1 is more likely to serve as the primary DNA glycosylase associated with dsDNA prior to replication fork unwinding. While it is possible that different results would be obtained with the oxidized base on the opposite strand, due to the inherent asymmetry of the replication fork, we think this is unlikely, especially as the results presented here indicate that it is the single- or double-strand nature of the DNA substrate that is the principal determining factor for activity. Therefore, it is tempting to speculate that the two DNA glycosylases scan DNA undergoing replication in a cooperative manner with the replisome, removing the same DNA lesions as the replication fork proceeds. This would indicate hNEIL3 as a vital DNA glycosylase in vivo associated with lesion excision during DNA unwinding, preventing the transfer of DNA mismatches and miscoded information downstream during mitosis to daughter cells. However further experimental work, which is ongoing, is required to validate this hypothesis.

## Figures and Tables

**Table 1:** Model DNA replication fork structures.

Fork−4	Fork	Fork+4
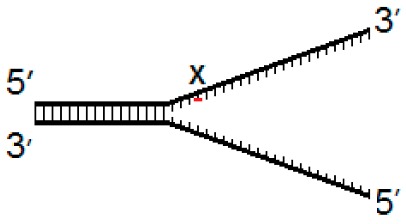	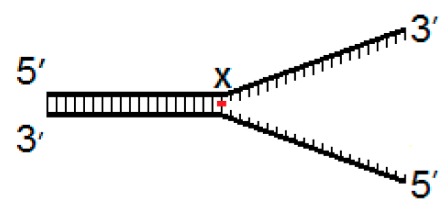	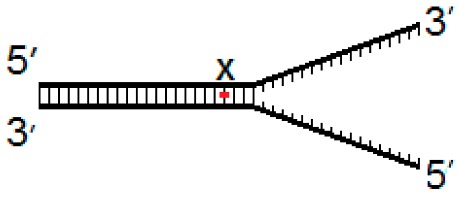

## Supplementary Materials

The following are available online at https://www.mdpi.com/2073-4425/10/4/315/s1, Figure S1: Anti-His western blot analysis of the flow-through (FT) and fractions 7–19 of hNEIL3^FL^ separated by FPLC on a Mono S 5/50 GL column.

## References

[1] Lindahl T. (1993). Instability and decay of the primary structure of DNA. Nature.

[2] Maynard S., Schurman S.H., Harboe C., de Souza-Pinto N.C., Bohr V.A. (2009). Base excision repair of oxidative DNA damage and association with cancer and aging. Carcinogenesis.

[3] Wallace S.S., Murphy D.L., Sweasy J.B. (2012). Base excision repair and cancer. Cancer Lett..

[4] Kelley M.R., Logsdon D., Fishel M.L. (2014). Targeting DNA repair pathways for cancer treatment: What’s new?. Future Oncol..

[5] Parsons J.L., Dianov G.L. (2013). Co-ordination of base excision repair and genome stability. DNA Repair (Amst.).

[6] Carter R.J., Parsons J.L. (2016). Base Excision Repair, a Pathway Regulated by Posttranslational Modifications. Mol. Cell Biol..

[7] Jacobs A.L., Schar P. (2012). DNA glycosylases: in DNA repair and beyond. Chromosoma.

[8] Regnell C.E., Hildrestrand G.A., Sejersted Y., Medin T., Moldestad O., Rolseth V., Krokeide S.Z., Suganthan R., Luna L., Bjørås M. (2012). Hippocampal adult neurogenesis is maintained by Neil3-dependent repair of oxidative DNA lesions in neural progenitor cells. Cell Rep..

[9] Wallace S.S. (2014). Base excision repair: a critical player in many games. DNA Repair (Amst.).

[10] Massaad M.J., Zhou J., Tsuchimoto D., Chou J., Jabara H., Janssen E., Glauzy S., Olson B.G., Morbach H., Ohsumi T.K. (2016). Deficiency of base excision repair enzyme NEIL3 drives increased predisposition to autoimmunity. J. Clin. Invest..

[11] Robson C.N., Hickson I.D. (1991). Isolation of cDNA clones encoding a human apurinic/apyrimidinic endonuclease that corrects DNA repair and mutagenesis defects in *E. coli* xth (exonuclease III) mutants. Nucleic Acids Res..

[12] Demple B., Herman T., Chen D.S. (1991). Cloning and expression of APE, the cDNA encoding the major human apurinic endonuclease: definition of a family of DNA repair enzymes. Proc. Natl. Acad. Sci. USA.

[13] Matsumoto Y., Kim K. (1995). Excision of deoxyribose phosphate residues by DNA polymerase b during DNA repair. Science.

[14] Sobol R.W., Horton J.K., Kühn R., Gu H., Singhal R.K., Prasad R., Rajewsky K., Wilson S.H. (1996). Requirement of mammalian DNA polymerase-beta in base-excision repair. Nature.

[15] Cappelli E., Taylor R., Cevasco M., Abbondandolo A., Caldecott K., Frosina G. (1997). Involvement of XRCC1 and DNA ligase III gene products in DNA base excision repair. J. Biol. Chem..

[16] Nash R.A., Caldecott K.W., Barnes D.E., Lindahl T. (1997). XRCC1 protein interacts with one of two distinct forms of DNA ligase III. Biochemistry.

[17] Dianov G., Price A., Lindahl T. (1992). Generation of single-nucleotide repair patches following excision of uracil residues from DNA. Mol. Cell. Biol..

[18] Zhou J., Chan J., Lambelé M., Yusufzai T., Stumpff J., Opresko P.L., Thali M., Wallace S.S. (2017). NEIL3 Repairs Telomere Damage during S Phase to Secure Chromosome Segregation at Mitosis. Cell Rep..

[19] Wiederhold L., Leppard J.B., Kedar P., Karimi-Busheri F., Rasouli-Nia A., Weinfeld M., Tomkinson A.E., Izumi T., Prasad R., Wilson S.H. (2004). AP endonuclease-independent DNA base excision repair in human cells. Mol. Cell.

[20] Liu M., Doublie S., Wallace S.S. (2013). Neil3, the final frontier for the DNA glycosylases that recognize oxidative damage. Mutat. Res..

[21] Liu M., Bandaru V., Holmes A., Averill A.M., Cannan W., Wallace S.S. (2012). Expression and purification of active mouse and human NEIL3 proteins. Protein Expr. Purif..

[22] Krokeide S.Z., Laerdahl J.K., Salah M., Luna L., Cederkvist F.H., Fleming A.M., Burrows C.J., Dalhus B., Bjørås M. (2013). Human NEIL3 is mainly a monofunctional DNA glycosylase removing spiroimindiohydantoin and guanidinohydantoin. DNA Repair (Amst.).

[23] Takao M., Oohata Y., Kitadokoro K., Kobayashi K., Iwai S., Yasui A., Yonei S., Zhang Q.M. (2009). Human Nei-like protein NEIL3 has AP lyase activity specific for single-stranded DNA and confers oxidative stress resistance in Escherichia coli mutant. Genes Cells.

[24] Martin P.R., Couve S., Zutterling C., Albelazi M.S., Groisman R., Matkarimov B.T., Parsons J.L., Elder R.H., Saparbaev M.K. (2017). The Human DNA glycosylases NEIL1 and NEIL3 Excise Psoralen-Induced DNA-DNA Cross-Links in a Four-Stranded DNA Structure. Sci. Rep..

[25] Zhou J., Fleming A.M., Averill A.M., Burrows C.J., Wallace S.S. (2015). The NEIL glycosylases remove oxidized guanine lesions from telomeric and promoter quadruplex DNA structures. Nucleic Acids Res..

[26] Zhou J., Liu M., Fleming A.M., Burrows C.J., Wallace S.S. (2013). Neil3 and NEIL1 DNA glycosylases remove oxidative damages from quadruplex DNA and exhibit preferences for lesions in the telomeric sequence context. J. Biol. Chem..

[27] Neurauter C.G., Luna L., Bjoras M. (2012). Release from quiescence stimulates the expression of human NEIL3 under the control of the Ras dependent ERK-MAP kinase pathway. DNA Repair (Amst.).

[28] Hazra T.K., Mitra S. (2006). Purification and characterization of NEIL1 and NEIL2, members of a distinct family of mammalian DNA glycosylases for repair of oxidized bases. Methods Enzymol..

[29] Hegde M.L., Hegde P.M., Bellot L.J., Mandal S.M., Hazra T.K., Li G.M., Boldogh I., Tomkinson A.E., Mitra S. (2013). Prereplicative repair of oxidized bases in the human genome is mediated by NEIL1 DNA glycosylase together with replication proteins. Proc. Natl. Acad. Sci. USA.

[30] Rangaswamy S., Pandey A., Mitra S., Hedge M.L. (2017). Pre-Replicative Repair of Oxidized Bases Maintains Fidelity in Mammalian Genomes: The Cowcatcher Role of NEIL1 DNA Glycosylase. Genes (Basel).

[31] Olsen M.B., Hildrestrand G.A., Scheffler K., Vinge L.E., Alfsnes K., Palibrk V., Wang J., Neurauter C.G., Luna L., Johansen J. (2017). NEIL3-Dependent Regulation of Cardiac Fibroblast Proliferation Prevents Myocardial Rupture. Cell Rep..

[32] Semlow D.R., Zhang J., Budzowska M., Drohat A.C., Walter J.C. (2016). Replication-Dependent Unhooking of DNA Interstrand Cross-Links by the NEIL3 Glycosylase. Cell.

[33] Couve-Privat S., Mace G., Rosselli F., Saparbaev M.K. (2007). Psoralen-induced DNA adducts are substrates for the base excision repair pathway in human cells. Nucleic Acids Res..

[34] Liu M., Bandaru V., Bond J.P., Jaruga P., Zhao X., Christov P.P., Burrows C.J., Rizzo C.J., Dizdaroglu M., Wallace S.S. (2010). The mouse ortholog of NEIL3 is a functional DNA glycosylase in vitro and in vivo. Proc. Natl. Acad. Sci. USA.

[35] Edmonds M.J., Carter R.J., Nickson C.M., Williams S.C., Parsons J.L. (2017). Ubiquitylation-dependent regulation of NEIL1 by Mule and TRIM26 is required for the cellular DNA damage response. Nucleic Acids Res..

[36] Klattenhoff A.W., Thakur M., Chu C.S., Ray D., Habib S.L., Kidane D. (2017). Loss of NEIL3 DNA glycosylase markedly increases replication associated double strand breaks and enhances sensitivity to ATR inhibitor in glioblastoma cells. Oncotarget.

