# Supplementary material for: The Biochemical Role of the Human NEIL1 and NEIL3 DNA Glycosylases on Model DNA Replication Forks
Source: Genes (Basel). 2019 Apr 23;10(4):315. doi: 10.3390/genes10040315 (PMC6523847; doi:10.3390/genes10040315)
Supplement: Supplementary file 1 [file genes-10-00315-s001.pdf]

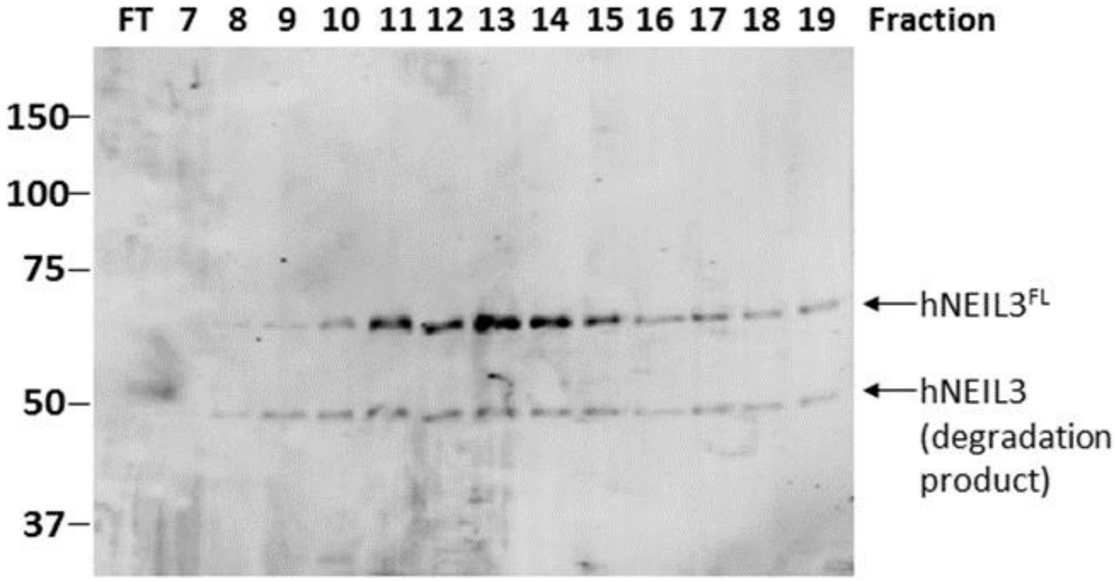

Figure S1. Anti-His Western blot analysis of the flow-through (FT) and fractions 7–19 of hNEIL3^{FL} separated by FPLC on a Mono S 5/50 GL column.
